# Beyond linearity: a threshold effect links serum creatinine to SIRI in osteoporotic fractures

**DOI:** 10.3389/fmed.2025.1710691

**Published:** 2025-12-18

**Authors:** Shuai Yuan, Ke Lu, Xiao-jie Zhou, Hao-tian Jiao, Yue-qin Guo, Chong Li

**Affiliations:** 1 Department of Orthopedics, Affiliated Kunshan Hospital of Jiangsu University, Suzhou, Jiangsu, China; 2 Department of Endocrinology, The Fifth People's Hospital of Kunshan, Suzhou, Jiangsu, China

**Keywords:** serum creatinine, systemic inflammatory response index, osteoporosis, fractures, inflammation

## Abstract

**Introduction:**

There is a scarcity of research examining the association between serum creatinine and the systemic inflammatory response index (SIRI) in individuals with osteoporotic fractures (OPF). Understanding this link may improve perioperative risk stratification and inflammatory management in OPF inpatients. Consequently, we explored the independent association between serum creatinine and SIRI and assessed potential non-linear or threshold effects in hospitalized OPF patients.

**Methods:**

A retrospective cross-sectional analysis was performed on 2,135 individuals with OPF who needed surgical intervention or inpatient care at affiliated Kunshan Hospital of Jiangsu University from January 2017 to August 2023. The exposure was serum creatinine and the outcome was SIRI. Covariates included age, sex, body mass index (BMI), hypertension, diabetes, heart disease, chronic kidney disease (CKD), alcohol consumption, smoking status, phosphorus, total cholesterol (TC), triglycerides, and aspartate aminotransferase (AST). Associations were estimated using multivariable linear regression and generalized estimating equations (GEE). Nonlinearity was examined using generalized additive models (GAMs) with smooth curve fitting and threshold (piecewise) analysis; univariable analyses were also performed.

**Results:**

After multivariable adjustment, serum creatinine was independently and positively associated with SIRI (*β*, 0.013; 95% CI, 0.004, 0.021, *p*-value < 0.01). GAM indicated a significant threshold effect (*p*-value for LRT = 0.02) with an inflection at 78 μmol/L. Above this threshold, the association strengthened (*β*, 0.033; 95% CI, 0.014, 0.052, *p*-value < 0.01).

**Conclusion:**

Among hospitalized patients with OPF, higher serum creatinine is associated with higher SIRI, with a clinically relevant threshold at 78 μmol/L. For creatinine values above this threshold, each 1 μmol/L increment corresponds to a 0.033 increase in SIRI. These findings support routine monitoring of creatinine, inflammatory status, and renal function in this population and warrant confirmation in prospective studies.

## 1 Introduction

Osteoporosis fracture (OPF) is a prevalent bone metabolic disorder characterized by reduced bone density, deterioration of bone microstructure, and loss of bone mass (1, 2). As the global aging population trend intensifies, its impact on public health is becoming increasingly severe (3, 4). Epidemiological data shows that there are approximately 8.9 million cases of osteoporotic fractures globally each year (5). In China, there were roughly 2.3 million osteoporotic fractures in 2010, with forecasts predicting an increase to 6 million by 2050 (6). This not only causes significant physical pain for patients but also imposes a substantial social and economic burden (7, 8). Recent evidence suggests that chronic inflammation raises the risk of osteoporosis and fractures (9). Thus, it is imperative to address the prevention of osteoporosis urgently.

Serum creatinine is a byproduct of the degradation of intracellular creatine phosphate and creatine precursors (10). In clinical settings, serum creatinine levels are commonly used to evaluate kidney function, and serum creatinine clearance is closely associated with the glomerular filtration rate. Research has shown that increased serum creatinine concentrations are linked to kidney disease, and deviations in serum creatinine levels can also impact clinical diagnosis and management (11–13). SIRI is a composite inflammatory marker derived from the counts of neutrophils (N), monocytes (M), and lymphocytes (L), used to assess the degree of systemic inflammatory response. The formula for calculating SIRI is generally $\frac{N\times M}{L}$ (14). This index can indicate the prognosis of patients with solid tumors, including pancreatic cancer, gastric cancer, and laryngeal cancer.

Recently, SIRI has garnered growing interest as a composite inflammatory marker that captures neutrophil, monocyte, and lymphocyte dynamics and has been linked to prognosis across diverse conditions (15, 16). Therefore, as a novel inflammatory biomarker, SIRI holds potential for use in clinical diagnosis and treatment. However, research on SIRI is still in evolving, and additional studies are required to validate its clinical application and significance in predicting the prognosis of various diseases.

Despite the widespread clinical use of serum creatinine as a renal function indicator and SIRI as a composite inflammatory biomarker, no prior study has specifically examined their relationship among hospitalized patients with OPF. This gap is clinically important for at least three reasons. First, OPF inpatients frequently experience heightened systemic inflammation in the perioperative and inpatient settings, and the interplay between inflammation and renal function may influence bone healing, infection risk, length of stay, and overall recovery. Second, identifying whether serum creatinine is associated with systemic inflammation (captured by SIRI) could help clinicians recognize patients at elevated inflammatory risk and anticipate complications. Third, if a clinically relevant threshold exists in the creatinine–SIRI association, it could provide actionable guidance for perioperative management, monitoring intensity, and individualized care strategies in this vulnerable population.

To address this gap, we investigated the independent association between baseline serum creatinine and SIRI in hospitalized OPF patients while rigorously adjusting for demographic, metabolic, and clinical covariates. In addition, we explored potential non-linearities using generalized additive models and performed threshold analyses to determine whether an inflection point exists. We hypothesized that higher serum creatinine would be associated with greater systemic inflammation, and that the relationship might exhibit a clinically meaningful threshold, thereby informing risk stratification and inpatient management.

## 2 Materials and methods

### 2.1 Research design and participants

**Figure 1:**
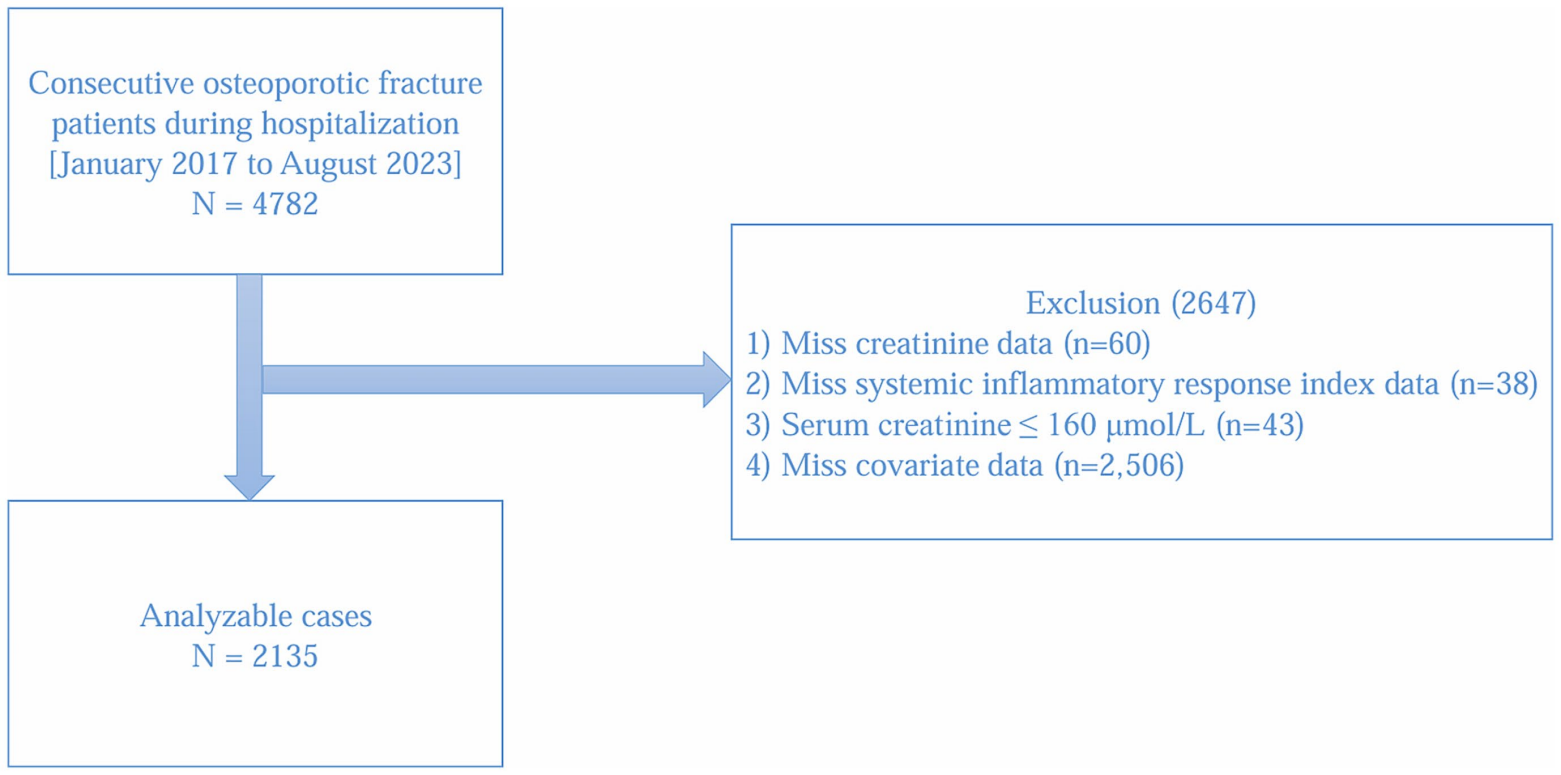
Study flow chart.

This was a retrospective study, utilizing data gathered from patient cases spanning from January 2017 to August 2023. The patient cases were obtained from the affiliated Kunshan Hospital of Jiangsu University in China. The research incorporated information from 4,782 patients with OPF who needed surgical intervention or hospitalization. OPF refers to a fracture resulting from reduced bone density, with the spine being the most frequent location for osteoporotic fractures. Fragile fracture refers to a fracture that occurs when a person falls from a standing height or lower, and common locations include the hip joint, wrist joint, and vertebrae. These fractures indicate potential osteoporosis, although some individuals may have normal or near normal bone density. This definition is supported by guidelines from the National Osteoporosis Foundation (NOF) and the International Osteoporosis Foundation (IOF) (17, 18). The identification of OPF was determined according to these criteria: (1) a bone mineral density (BMD) T-score of −2.5 or lower; (2) imaging assessments, such as dual-energy X-ray absorptiometry (DEXA) scans, which accurately evaluate bone density and identify potential fragility fractures, (3) osteoporosis-related fractures are primarily classified under ICD-10 codes ‘M80’ and ‘M81’; and (4) pathological assessment showing reduced bone density and osteoporosis (19, 20). Inclusion criteria: (1) age ≥50 years; (2) hospitalized for OPF requiring surgical intervention or inpatient management; (3) availability of baseline laboratory assessments (serum creatinine and differential blood counts for SIRI) within the first 3 days of hospitalization; (4) OPF attributable to osteoporosis, defined by a BMD T-score ≤−2.5 and/or pathological assessment indicating reduced bone density and osteoporosis. Exclusion criteria: (1) missing serum creatinine data (*n* = 60); (2) missing SIRI data (*n* = 38); (3) markedly aberrant serum creatinine values, exceeding the normal range by >20% (serum creatinine ≥160 μmol/L) (n = 43) (21); (4) missing key covariate data (*n* = 2,506). No upper age limit was set. Comorbidities (e.g., hypertension, diabetes, heart disease, CKD) were not used as exclusion criteria and were instead adjusted as covariates in multivariable models. As depicted in Figure 1, following the application of selection criteria, the study comprised a total of 2,135 hospitalized patients with OPF.

This is a retrospective observational study. We received ethical approval from the Affiliated Kunshan Hospital of Jiangsu University (approval number 2024-03-053-K01), and was compliant with the Declaration of Helsinki. As this was an observational study and data were gathered anonymously, written informed consent was not required for these analyses.

### 2.2 Sample size determination

This retrospective study included all consecutive eligible inpatients with OPF admitted between January 2017 and August 2023. No formal *a priori* sample size calculation was performed. The final analytic cohort (*N* = 2,135) reflects a complete-case approach and was used consistently across all models (Models 1–3). We relied on precision-based justification, presenting effect estimates with 95% confidence intervals to demonstrate the adequacy of the sample for the planned multivariable analyses.

### 2.3 Dependent variables

In this study, the dependent variable was SIRI, calculated using the formula N × M/L (SIRI = $\frac{N\times M}{L}$) (14). The assessment technique utilized flow cytometry with a Sysmex XN-10 (B4) hematology analyzer, where the levels of neutrophils, monocytes, and lymphocytes were measured using nuclear staining. The absolute counts of neutrophils, monocytes, and lymphocytes were obtained from fasting blood samples collected from hospitalized patients. These counts were analyzed using standard complete blood count (CBC) testing.

### 2.4 Independent variables

In this research, the independent variable was serum creatinine levels. Serum creatinine levels were assessed using an automatic biochemical analyzer (AU480, Beckman Coulter), and were quantified after fasting within the initial 3 days of hospitalization (22).

### 2.5 Covariates

The covariates included in the study were age, gender, BMI, hypertension, diabetes, heart disease, CKD, alcohol consumption, smoking status, phosphorus, TC, AST. All clinical variables were measured after fasting during the first 3 days of hospitalization.

### 2.6 Statistics

All assessment were obtained with the identical device and by the same skilled technician adhering to uniform procedures. Results were presented as mean ± standard deviation (SD), median (Q1, Q3), or percentage frequency (%) for both continuous and categorical variables, as applicable. Univariate analysis of categorical variables was performed using Pearson’s chi-square test or Fisher’s exact test. Continuous variables were analyzed using either *t*-tests or Mann–Whitney *U* tests, based on whether the data adhered to a normal or non-normal distribution. Linear multivariate regression models were used to evaluate the association among serum creatinine concentrations and SIRI in hospitalized patients with OPF.

GEE with proper covariate adjustments were utilized to investigate the independent association between serum creatinine and SIRI in hospitalized patients with OPF. The models developed comprised a basic model (Model 1), a lightly adjusted model (Model 2) that accounted for age, gender, and BMI, and a comprehensively adjusted model (Model 3) which included further adjustments for age, gender, BMI, hypertension, diabetes, heart disease, CKD, alcohol consumption, smoking status, phosphorus, TC, triglycerides, and AST. All three models were estimated using the same complete-case analytic cohort (*N* = 2,135), as defined in the study flowchart (Figure 1). Initially, variance inflation factor analysis was conducted to identify multicollinearity among the covariates, and the covariates were chosen for adjustment based on the subsequent criteria: (1) When adding or removing a covariates in the basic or comprehensive model, a change in the matched odds ratio (OR) of ≥10% was noted, and (2) covariates that satisfied criterion 1 or exhibited a *p*-value < 0.1 in the univariate analyses (23).

GAMs were used to detect potential non-linear associations. When such relationships were evident, piecewise linear regression models were employed to identify threshold effects of the smoothed curves. Where these curves exhibited clear inflection points, a recursive method using maximum likelihood models was used to automatically calculate the breakpoints. Subgroup analyses were conducted by categorizing patients based on relevant covariates to verify the consistency of the analyses and examine their differences across various patient categories. Additionally, likelihood ratio tests (LRTs) were employed to evaluate interactions within these subgroups.

All statistical analyses were conducted using Empower Stats (X&Y Solutions, Inc., Boston, MA, USA).^1^ R software version 3.6.3 was also applied.^2^ A *p*-value under 0.05 was deemed statistically significant.

## 3 Results

### 3.1 Patient characteristic

Table 1 presents the baseline features of 2,135 hospitalized patients with OPF from January 2017 to August 2023, categorized into four groups according to their serum creatinine concentrations. These patients (67.12% females and 32.88% males) had a mean age of 69.06 ± 11.15 years, mean serum creatinine level of 63.23 ± 18.04 μmol/L, and mean SIRI of 3.22 ± 3.71. Stratification by serum creatinine levels (<51 μmol/L, 51–60 μmol/L, 61–71 μmol/L, >71 μmol/L) revealed significant differences among the groups in terms of SIRI, age, gender, diabetes, smoking status, AST and TC.

**Table 1:** Patient characteristics categorized by serum creatinine quartiles.

Characteristics	Total	Mean + SD/*N* (%)				*p*-value	*p*-value*
		Q1 (<51 μmol/L)	Q2 (51–60 μmol/L)	Q3 (61–71 μmol/L)	Q4 (>71 μmol/L)		
*N*	2,135	518	541	507	569		
SIRI	3.22 ± 3.71	2.97 ± 3.56	3.24 ± 4.66	3.32 ± 3.39	3.37 ± 3.23	0.31	<0.01
Age 4 quantiles, y						0.01	–
Q1 (<60y)	489 (22.90%)	143 (27.61%)	129 (23.84%)	106 (20.91%)	111 (19.52%)		
Q2 (60–68y)	523 (24.50%)	127 (24.51%)	136 (25.14%)	134 (26.43%)	126 (22.14%)		
Q3 (69–78y)	581 (27.21%)	140 (27.03%)	149 (27.54%)	133 (26.23%)	159 (27.94%)		
Q4 (>78y)	542 (25.29%)	108 (20.85%)	127 (23.48%)	134 (26.43%)	173 (30.40%)		
BMI categorical, *N* (%)						0.48	–
<24 kg/m^2^	1,302 (60.98%)	309 (59.65%)	327 (60.44%)	301 (59.37%)	365 (64.15%)		
24–27 kg/m^2^	685 (32.09%)	174 (33.59%)	181 (33.46%)	164 (32.35%)	166 (29.17%)		
≥28 kg/m^2^	148 (6.93%)	35 (6.76%)	33 (6.10%)	42 (8.28%)	38 (6.68%)		
Gender, *N* (%)						<0.01	–
Female	1,433 (67.12%)	300 (57.92%)	354 (65.43%)	353 (69.63%)	426 (74.87%)		
Male	702 (32.88%)	218 (42.08%)	187 (34.57%)	154 (30.37%)	143 (25.13%)		
Hypertension, *N* (%)						0.80	–
No	1,847 (86.51%)	448 (86.49%)	473 (87.43%)	440 (86.79%)	486 (85.41%)		
Yes	288 (13.49%)	70 (13.51%)	68 (12.57%)	67 (13.21%)	83 (14.59%)		
Diabetes, *N* (%)						0.03	–
No	2,055 (96.25%)	493 (95.17%)	518 (95.75%)	485 (95.66%)	559 (98.24%)		
Yes	80 (3.75%)	25 (4.83%)	23 (4.25%)	22 (4.34%)	10 (1.76%)		
Heart diseases, *N* (%)						0.63	–
No	2,085 (97.66%)	505 (97.49%)	527 (97.41%)	499 (98.42%)	554 (97.36%)		
Yes	50 (2.34%)	13 (2.51%)	14 (2.59%)	8 (1.58%)	15 (2.64%)		
CKD, *N* (%)						0.54	–
No	2,133 (99.91%)	517 (99.81%)	541 (100.00%)	506 (99.80%)	569 (100.00%)		
Yes	2 (0.09%)	1 (0.19%)	0 (0.00%)	1 (0.20%)	0 (0.00%)		
Alcohol consumption, *N* (%)						0.12	–
No	2,030 (95.08%)	484 (93.44%)	516 (95.38%)	490 (96.65%)	540 (94.90%)		
Yes	105 (4.92%)	34 (6.56%)	25 (4.62%)	17 (3.35%)	29 (5.10%)		
Smoking status, *N* (%)						0.04	–
No	1,978 (92.65%)	465 (89.77%)	504 (93.16%)	476 (93.89%)	533 (93.67%)		
Yes	157 (7.35%)	53 (10.23%)	37 (6.84%)	31 (6.11%)	36 (6.33%)		
AST, U/L	24.94 ± 14.94	24.25 ± 13.21	24.34 ± 13.74	25.48 ± 14.54	25.68 ± 18.27	0.27	0.01
Serum phosphorus, mmol/L	1.06 ± 0.20	1.07 ± 0.19	1.07 ± 0.20	1.05 ± 0.21	1.06 ± 0.22	0.51	0.42
TC, mmol/L	4.23 ± 0.92	4.35 ± 0.91	4.29 ± 0.91	4.24 ± 0.92	4.06 ± 0.94	<0.01	<0.01
Triglycerides, mmol/L	1.23 ± 0.96	1.20 ± 0.95	1.28 ± 1.07	1.25 ± 0.96	1.20 ± 0.85	0.47	0.49

SD, standard deviation; Q1, first quartile; Q2, second quartile; Q3, third quartile; Q4, fourth quartile; SIRI, systemic inflammation response index; BMI, body mass index; CKD, chronic kidney disease; AST, aspartate aminotransferase; TC, total cholesterol.

*p-value: Kruskal Wallis Rank Test for continuous variables, Fisher Exact for categorical variables with Expects < 10.

### 3.2 Univariate analyses of factors associated with SIRI

In univariate analysis, significant associations were observed between SIRI and variables including gender (*p* = 0.04), phosphorus (*p* < 0.01), TC (*p* < 0.01), triglycerides (*p* < 0.01), AST (*p* < 0.01), and serum creatinine (*p* < 0.01) (Table 2).

**Table 2:** Univariate analyses of factors associated with SIRI.

Characteristics	Statistics	*β*^a^ (95% CI) *p*-value
Age 4 quantiles, y		
Q1 (<60y)	489 (22.90%)	Reference
Q2 (60–68y)	523 (24.50%)	−0.199 (−0.662, 0.263) 0.40
Q3 (69–78y)	581 (27.21%)	0.004 (−0.447, 0.456) 0.99
Q4 (>78y)	542 (25.39%)	0.063 (−0.395, 0.522) 0.79
Gender, *N* (%)		
Female	1,433 (67.12%)	Reference
Male	702 (32.88%)	0.352 (0.014, 0.690) 0.04
BMI categorical, *N* (%)		
<24 kg/m^2^	1,302 (60.98%)	Reference
24–27 kg/m^2^	685 (32.09%)	−0.063 (−0.410, 0.284) 0.72
≥28 kg/m^2^	148 (6.93%)	−0.679 (−1.317, −0.042) 0.04
Hypertension, *N* (%)		
No	1,847 (86.51%)	Reference
Yes	288 (13.49%)	−0.446 (−0.911, 0.020) 0.06
Diabetes, *N* (%)		
No	2,055 (96.25%)	Reference
Yes	80 (3.75%)	−0.554 (−1.392, 0.284) 0.20
Heart diseases, *N* (%)		
No	2,085 (97.66%)	Reference
Yes	50 (2.34%)	−0.330 (−1.382, 0.723) 0.54
CKD, *N* (%)		
No	2,133 (99.91%)	Reference
Yes	2 (0.09%)	0.383 (−4.819, 5.584) 0.89
Alcohol consumption, *N* (%)		
No	2,030 (95.08%)	Reference
Yes	105 (4.92%)	0.365 (−0.371, 1.101) 0.33
Smoking status, *N* (%)		
No	1,978 (92.65%)	Reference
Yes	157 (7.35%)	0.467 (−0.143, 1.076) 0.13
Serum phosphorus, mmol/L	1.06 ± 0.20	−4.504 (−5.263, −3.746) < 0.01
TC, mmol/L	4.23 ± 0.92	−0.522 (−0.693, −0.352) < 0.01
Triglycerides, mmol/L	1.23 ± 0.96	−0.2660 (−0.432, −0.100) < 0.01
AST, U/L	24.95 ± 15.15	0.042 (0.032, 0.053) < 0.01
Serum creatinine, μmol/L	63.23 ± 18.04	0.016 (0.007, 0.024) < 0.01
Serum creatinine 4 quartiles, μmol/L		
Q1 (<51 μmol/L)	518 (24.26%)	Reference
Q2 (51–60μmol/L)	541 (25.34%)	0.275 (−0.176, 0.727) 0.23
Q3 (61–71μmol/L)	507 (23.75%)	0.350 (−0.109, 0.810) 0.13
Q4 (>71 μmol/L)	569 (26.65%)	0.404 (−0.043, 0.850) 0.08

a Dependent variable SIRI, based on univariate analyses of SIRI.

SIRI, systemic inflammation response index; CKD, chronic kidney disease; BMI, body mass index; TC, total cholesterol; AST, aspartate aminotransferase.

### 3.3 Exploration of the association between serum creatinine and SIRI

Table 3 utilizes three different models to investigate the relationship between serum creatinine and SIRI in hospitalized patients with OPF. In Model 1 (without adjustments), a significant relationship between the variables was identified (*β*, 0.016; 95% CI, 0.007, 0.024, *p*-value < 0.01). Model 2, which adjusted for age, gender, and BMI, demonstrated a comparable relationship (*β*, 0.017; 95% CI, 0.008, 0.025, *p*-value < 0.01). Model 3, which accounted for factors such as age, gender, BMI, hypertension, diabetes, heart diseases, CKD, alcohol consumption, smoking status, phosphorus, TC, triglycerides and AST likewise revealed a noteworthy connection (*β*, 0.013; 95% CI, 0.004, 0.021, *p*-value < 0.01). The *β* coefficient represents the variation in SIRI for each unit increase in serum creatinine level. In all models, a significant positive association was found between serum creatinine and SIRI, indicating that higher levels of serum creatinine are linked to increased SIRI values.

**Table 3:** Relationship between serum creatinine and SIRI across various models.

Outcome	Model 1^a^	Model 2^b^	Model 3^c^
	N = 2,135	N = 2,135	N = 2,135
	*β* (95% CI)	*β* (95% CI)	*β* (95% CI)
	*p*-value	*p*-value	*p*-value
Cr per 1 μmol/L increase	0.016 (0.007, 0.024) < 0.01	0.017 (0.008, 0.025) < 0.01	0.013 (0.004, 0.021) < 0.01

a No adjustment.

b Adjusted for age; gender; BMI.

c Adjusted for age; gender; BMI; hypertension; diabetes; heart diseases; CKD; alcohol consumption; smoking status; serum phosphorus; total cholesterol, triglycerides; AST.

SIRI, systemic inflammation response index; BMI, body mass index; CKD, chronic kidney disease; AST, aspartate aminotransferase.

Clinically, these findings imply that higher serum creatinine levels may contribute to increased inflammation in patients, possibly indicating underlying health conditions or the severity of disease. These results highlight the need to factor in serum creatinine levels when evaluating inflammation and overall health outcomes in both medical practice and scientific investigation.

### 3.4 Spline smoothing plot and threshold analyses

**Figure 2:**
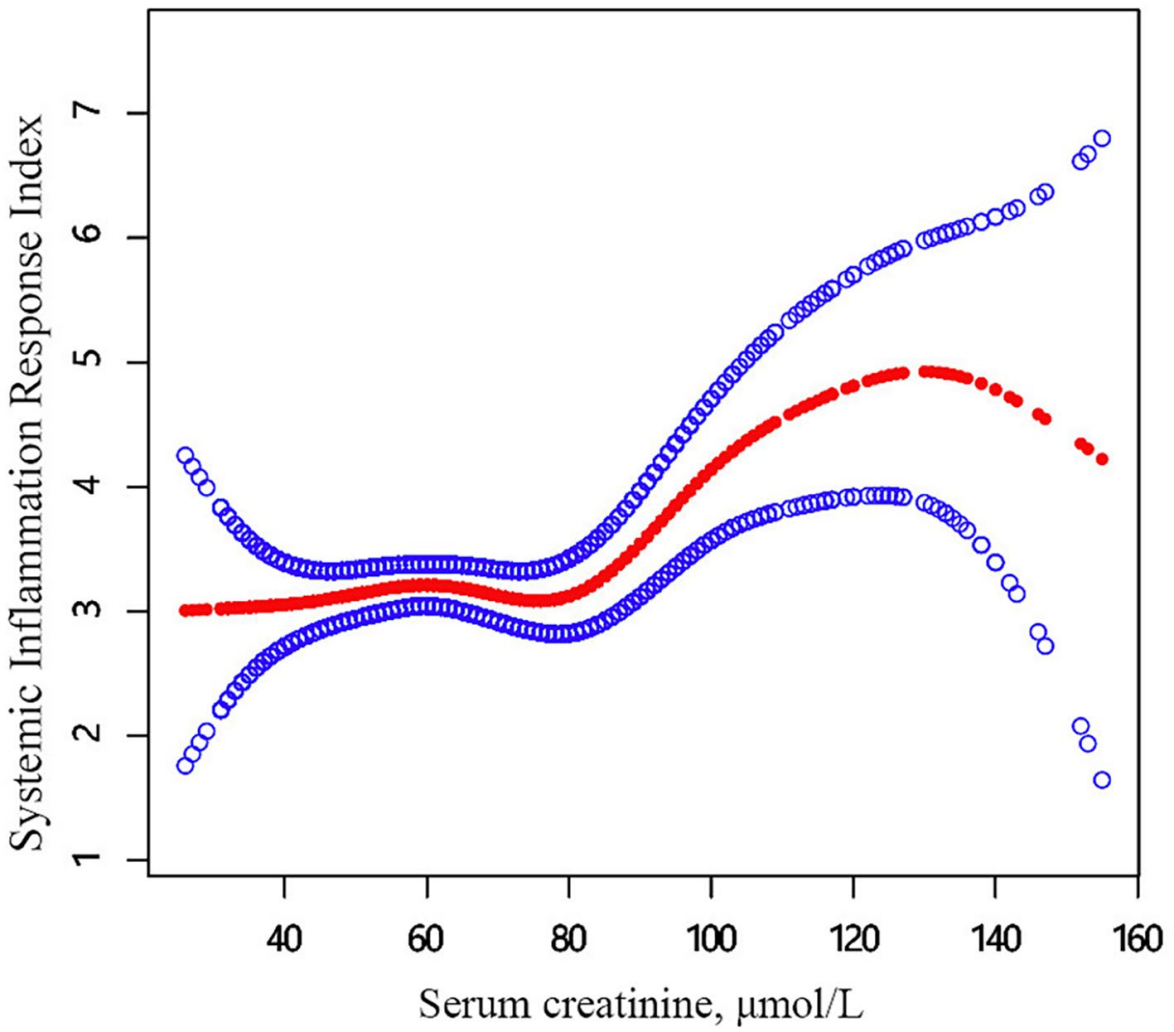
The relationship between serum creatinine and SIRI. Adjusted smoothed curves corresponding to the relationship between serum creatinine and SIRI. A generalized additive model revealed a thresholded non-linear relationship between serum creatinine and SIRI in hospitalized patients with OPF. The upper and lower curves represent the range of the 95% confidence interval, and the middle curve represents the association between serum creatinine and SIRI. Models were adjusted for age; gender; BMI; hypertension; diabetes; heart diseases; CKD; alcohol consumption; smoking status; serum phosphorus; total cholesterol, triglycerides; AST. The red curve in Model 3 exhibited an inflection point (K) at 78 μmol/L. SIRI, systemic inflammation response index; BMI, body mass index; CKD, chronic kidney disease; AST, aspartate aminotransferase.

Firstly, the curve relationship between serum creatinine and SIRI in hospitalized patients with OPF was depicted using graphical methods (Figure 2). Secondly, GAM analysis, which accounted for age, gender, BMI, hypertension, diabetes, heart disease, CKD, alcohol consumption, smoking status, phosphorus, TC, triglycerides and AST identified a nonlinear relationship between serum creatinine and SIRI (Table 4). Segmented linear regression models were employed to verify the threshold nonlinear relationship. A threshold point for serum creatinine was identified at 78 μmol/L. Below this level (<78 μmol/L), no notable relationship was found between serum creatinine and SIRI. Conversely, above this level (>78 μmol/L), SIRI markedly increased (*β*, 0.033; 95% CI, 0.014, 0.052, *p*-value < 0.01). These results highlight a strong link between serum creatinine levels above 78 μmol/L and increased systemic inflammation, while no significant connection is observed below this level.

**Table 4:** Threshold effect analysis investigating the relationship between serum creatinine and SIRI.

Outcome	Model 3^a^
	*β* (95% CI)
	*p*-value
Model A^b^	
One line effect	0.013 (0.004, 0.021) < 0.01
Model B^c^	
Cr turning Point (K), μmol/L	78
<K	0.001 (−0.013, 0.014) 0.94
>K	0.033 (0.014, 0.052) < 0.01
SIRI value at K	3.24 (2.94, 3.55)
LRT test^d^	0.02

a Adjusted for age; gender; BMI; hypertension; diabetes; heart diseases; CKD; alcohol consumption; smoking status; serum phosphorus; total cholesterol; triglycerides; AST.

b Linear analysis, A *p*-value < 0.05 suggests a linear relationship.

c Non-linear analysis.

d A *p*-value < 0.05 indicates that Model B differs significantly different from Model A, suggesting a non-linear relationship.

SIRI, systemic inflammation response index; BMI, body mass index; CKD, chronic kidney disease; AST, aspartate aminotransferase; LRT, logarithmic likelihood ratio test.

### 3.5 Sensitivity analyses

To confirm the robustness of Model 3, additional segment analyses were performed. Hospitalized patients with OPF were categorized according to age, gender, BMI, phosphorus, TC, triglycerides and AST, with adjustment for remaining covariates (Supplementary Table S1). The findings showed that all segments were stable, and the partitioning within each segment remained consistent.

Supplementary Table S2 presents the regression coefficients (*β*) and associated *p*-values for various creatinine thresholds analyzed in the study. The models assess the impact of creatinine levels, quantified as an increase of 1 μmol/L, on the outcomes of interest. Model 1 is adjusted for age, gender, and BMI, while Model 2 incorporates additional covariates such as hypertension, diabetes, heart disease, CKD, substance use, and lipid parameters. All models indicate statistically significant results (*p* < 0.01) supporting a robust relationship between elevated creatinine levels and the studied health outcomes.

To address potential confounding effects, we excluded patients with acute illnesses or stress-related inflammatory conditions from our analysis. Acute illness was defined based on specific clinical diagnoses and laboratory indicators, ensuring that only patients with stable chronic conditions were included (Supplementary Table S3). The incorporation of the frailty index revealed a significant interaction with serum creatinine and SIRI levels, suggesting that frailty may influence the observed relationship. Our sensitivity analysis demonstrated a robust association between serum creatinine and SIRI with patient outcomes (Supplementary Table S3). In the primary analysis, serum creatinine and SIRI exhibited a *β* coefficient of 0.013 (95% CI: 0.004–0.021, *p* < 0.01). This coefficient remained consistent at 0.014 (95% CI: 0.005–0.023, *p* < 0.01) upon including the frailty index. Furthermore, after excluding individuals with acute illnesses or stress-related inflammatory states, the *β* coefficient persisted at 0.013 (95% CI: 0.006–0.020, *p* < 0.01), confirming the reliability of our findings. Stratified analysis revealed even stronger associations in serum creatinine over 78 μmol/L, with *β* coefficients of 0.033 (95% CI: 0.014–0.052) and 0.035 (95% CI: 0.015–0.054). These results underscore the significance and consistency of serum creatinine and SIRI in the prognostic evaluation of patients with cardiovascular disease.

## 4 Discussion

This retrospective cross-sectional study investigated the relationship between serum creatinine and SIRI. Our findings initially identified the threshold effect between serum creatinine and SIRI in hospitalized patients with OPF in Kunshan, China. The piecewise linear regression model identified a threshold of 78 μmol/L for serum creatinine. When serum creatinine levels were below this threshold (<78 μmol/L), no significant association was found between SIRI and serum creatinine concentrations. In contrast, when serum creatinine concentrations exceeded the threshold (>78 μmol/L), there was a notable increase in SIRI (*β*, 0.033; 95% CI, 0.014, 0.052, *p*-value < 0.01). Exceeding this value has significant implications for assessing renal impairment and systemic inflammation, as elevated serum creatinine levels correlate strongly with increased SIRI, indicating a heightened inflammatory state that could adversely affect clinical outcomes. This discovery offers a data-driven basis for managing serum creatinine concentrations, inflammatory status, and renal function in hospitalized patients with OPF.

The connection between serum creatinine and SIRI has been infrequently studied, with the existing literature presenting limited results (24–27). However, several studies have found a positive relationship between these two parameters, which is consistent with the present findings (24–27). Prior research has indicated that higher SIRI levels might be associated with increased creatine kinase concentrations and greater metabolic turnover, leading to elevated serum creatinine concentrations (24–26). Additionally, SIRI-induced capillary leakage and a reduced effective circulating volume can activate the sympathetic nervous system, resulting in renal artery constriction and a decreased glomerular filtration rate, which in turn contributes to the increase in serum creatinine concentrations (27). Additionally, an increase in SIRI can negatively affect renal function and boost the production of pro-inflammatory mediators, potentially further raising serum creatinine concentrations (28). Collectively, these findings provide plausible mechanistic insights into the positive association observed between serum creatinine and SIRI. The heightened metabolic activity, altered renal hemodynamics, and inflammation-driven changes in renal function associated with elevated SIRI appear to be critical factors underlying the observed relationship between these two parameters. Understanding the intricate interaction between systemic inflammation, indicated by SIRI, and creatinine metabolism could have significant clinical consequences. Additionally, comparing SIRI’s relationship with serum creatinine to that of other inflammatory markers, such as CRP and NLR, may highlight SIRI’s relative utility as a biomarker in these patients. Further research is warranted to delineate the precise pathways linking these two factors and to explore their potential utility as integrated biomarkers for the assessment and management of various disease states.

Serum creatinine is a well-recognized byproduct of intracellular creatine and phosphocreatine, and has been thoroughly investigated as a biomarker across numerous medical conditions, such as end-stage renal disease, cardiovascular disorders, diabetes, and muscular dystrophy (29–33). Current evidence indicates that serum creatinine primarily affects BMD by acting as a scavenger of hydroxyl radicals, thereby mitigating oxidative stress and regulating bone resorption (17). Elevated serum creatinine levels have been associated with reduced BMD or heightened bone resorption (17), whereas lower serum creatinine concentrations have also been linked to diminished BMD (34). Creatinine, a byproduct of metabolism of creatine and phosphocreatine, plays a crucial role in maintaining cellular energy homeostasis and redox balance. Its role as an effective scavenger of hydroxyl radicals helps reduce oxidative stress, a major factor in the development of osteoporosis and other bone diseases (17). High serum creatinine levels, commonly seen in conditions like end-stage renal disease, have been linked to reduced BMD and enhanced bone resorption, likely due to the harmful impact of oxidative stress on bone metabolism (17). On the other hand, low serum creatinine levels, which can arise from conditions such as muscle wasting or malnutrition, have also been linked to decreased BMD, highlighting the intricate relationship between creatinine metabolism and bone integrity (34). These results highlight the significance of serum creatinine as a possible biomarker and treatment target in relation to bone integrity and disorders. Additional studies are needed to clarify the exact mechanisms through which creatinine metabolism affects bone equilibrium and to investigate the clinical significance of these connections for the prevention and management of bone diseases.

SIRI is an inflammatory marker based on the counts of neutrophils, monocytes, and lymphocytes (9). SIRI has been examined as a marker in a range of conditions, including skeletal disorders, cancer, COVID-19, cardiovascular disease, thyroid disorders, pancreatic disease, and gallbladder disease (14, 35–38). Clinicians can utilize SIRI to evaluate a patient’s level of inflammation, monitor disease progression and prognosis, and inform suitable treatment strategies. Research by Ha Neul Kim and associates revealed that in patients with axial spondyloarthritis (axSpA), regardless of their BMD status, the average scores for acute inflammation and structural damage were elevated in the low BMD group across six slices. Additionally, the mean depth score was greater among those with low BMD (39). This research indicates that immune cells activated at inflammation sites release various cytokines, which enhance bone resorption, resulting in bone degradation, osteitis, and both localized and systemic bone deterioration. Certain researchers have discovered that inflammation can impact osteoporosis not only through cytokine profiles but also by influencing factors involved in bone metabolism (40–42). Patil et al. noted that inflammatory diseases frequently cause elevated levels of inflammatory cytokines in both bone tissue and serum, which leads to heightened osteoclast activity and subsequent bone deterioration and osteoporosis (43). Moreover, Khinda et al. discovered that chronic inflammation can decrease bone density, and elevated concentrations of the inflammatory marker C-reactive protein (CRP) are inversely related to bone density, indicating a direct connection between inflammation and osteoporosis (44). These findings offer robust evidence of the association between inflammation and osteoporosis. Thus, a novel inflammatory marker, SIRI, may be employed to evaluate the overall inflammatory condition in populations at risk for osteoporosis. When SIRI levels are elevated, prompt action to lower the inflammation is essential to prevent osteoporosis.

The discovery of a positive association and threshold effect between serum creatinine and SIRI provides evidence-based medical guidance for the management of serum creatinine, inflammatory status, and renal function in hospitalized patients with OPF. Firstly, for surgical patients with OPF, maintaining serum creatinine levels below 78 μmol/L may reduce the inflammatory state during or around the surgical period. When serum creatinine levels surpass 78 μmol/L, the SIRI index notably rises, which may impair the management of inflammation in surgical patients with OPF. Additionally, excessively elevated SIRI levels suggest a severe systemic inflammatory state, which could affect renal function. Hence, keeping SIRI below 3.24 might offer protective benefits for the kidneys. Moreover, managing the inflammatory condition could also aid in alleviating inflammatory osteoporosis. In conclusion, keeping serum creatinine levels under 78 μmol/L, avoiding excessively high SIRI levels, and managing inflammation may help regulate the inflammatory condition and potentially enhance inflammatory osteoporosis outcomes.

This is the first study in China to conduct an epidemiological investigation on the independent association between serum creatinine and SIRI in hospitalized patients with OPF. Our research may contribute to the clinical practice for hospitalized patients with OPF. SIRI is a crucial marker for evaluating inflammation progression and significantly contributes to the development of osteoporosis (45). The identified threshold effect between serum creatinine and SIRI suggests that when serum creatinine levels exceed 78 μmol/L, SIRI rises significantly. Therefore, in clinical practice, controlling serum creatinine levels within the optimal range for hospitalized patients with OPF can help lower their SIRI levels. Grasping this is crucial for the clinical diagnosis and management of hospitalized patients with OPF. Moreover, this discovery can help mitigate the risk of inflammatory osteoporosis by targeting and reducing inflammation levels, and it can also aid in developing suitable treatment plans for individuals with inflammatory osteoporosis.

This analysis has several limitations. Firstly, as this is a cross-sectional study, it only shows an association between serum creatinine and SIRI, without establishing a causal link. Secondly, although the study adjusted for certain variables, it did not consider potential confounding factors like variations in blood parameters and residual effects of medications in the analysis. To gain deeper insight into the relationship between serum creatinine and SIRI, additional experimental designs and stratified cohort studies are necessary, along with the consideration of new potential confounding factors. Thirdly, the study focused on hospitalized patients with OPF aged 50 and older, which means the findings may not be relevant to individuals under 50 years of age. Fourth, as this was a single-center study, the results may not be applicable to other ethnic groups. Therefore, additional research is needed, including more extensive analyses covering a wider range of ages and ethnicities, as well as multi-center randomized controlled studies, to validate the findings of the current study.

## 5 Conclusion

In summary, the findings of this research indicate a threshold effect between serum creatinine and SIRI in hospitalized patients with OPF. When serum creatinine exceeds 78 μmol/L, there is a strong positive association between serum creatinine and SIRI, leading to a notable increase in SIRI. Identifying the threshold effect between serum creatinine and SIRI offers a data-driven justification for managing serum creatinine levels, inflammation, and kidney function in hospitalized patients with OPF. This discovery can assist clinicians in enhancing the care of hospitalized patients with OPF by recommending suitable monitoring and management of serum creatinine and SIRI levels to reduce the negative effects of inflammation and preserve renal function. Additionally, this finding can aid in the clinical evaluation and diagnosis of individuals at risk for osteoporosis, and guide the implementation of effective measures to prevent and mitigate osteoporosis risk. Nevertheless, more detailed analyses and cohort studies are required to validate the accuracy of the current study’s findings.

## Data Availability

The original contributions presented in the study are included in the article/Supplementary material, further inquiries can be directed to the corresponding author.

## Footnotes

1
www.empowerstats.com

2
http://www.r-project.org
