# Supplementary material for: Beyond linearity: a threshold effect links serum creatinine to SIRI in osteoporotic fractures
Source: Front Med (Lausanne). 2025 Dec 18;12:1710691. doi: 10.3389/fmed.2025.1710691 (PMC12756718; doi:10.3389/fmed.2025.1710691)
Supplement: Supplementary file 1 [file Table_1.docx]

**Table S1.** Subgroup analyses investigating the association between serum creatinine and SIRI

|  | N | β (95% CI) *P*-value | |
| --- | --- | --- | --- |
| Age tertile, y |  | |  |
| Low (< 63y) | 652 | | 0.008 (-0.009, 0.025) 0.36 |
| Middle (63-74y) | 745 | | 0.021 (0.006, 0.035) 0.01 |
| High (> 74y) | 738 | | 0.016 (0.001, 0.030) 0.03 |
| Gender, N (%) |  | |  |
| Female | 1433 | | 0.019 (0.008, 0.030) < 0.01 |
| Male | 702 | | 0.012 (-0.003, 0.028) 0.12 |
| BMI tertile, kg/m^2^ |  | |  |
| < 24 | 1302 | | 0.009 (-0.002, 0.020) 0.09 |
| 24-27 | 685 | | 0.029 (0.012, 0.045) < 0.01 |
| ≥ 28 | 148 | | 0.023 (-0.011, 0.057) 0.18 |
| Hypertension, N (%) |  | |  |
| No | 1847 | | 0.017 (0.007, 0.027) < 0.01 |
| Yes | 288 | | 0.009 (-0.014, 0.032) 0.46 |
| Diabetes, N (%) |  | |  |
| No | 2055 | | 0.015 (0.007, 0.024) < 0.01 |
| Yes | 80 | | 0.012 (-0.040, 0.063) 0.65 |
| Heart diseases, N (%) |  | |  |
| No | 2085 | | 0.015 (0.006, 0.024) < 0.01 |
| Yes | 50 | | 0.033 (-0.024, 0.090) 0.26 |
| CKD, N (%) |  | |  |
| No | 2133 | | 0.016 (0.007, 0.024) < 0.01 |
| Yes | 2 | | 0.070 (-0.288, 0.427) 0.70 |
| Alcohol consumption, N (%) |  | |  |
| No | 2030 | | 0.016 (0.007, 0.025) < 0.01 |
| Yes | 105 | | 0.013 (-0.023, 0.049) 0.48 |
| Smoking status, N (%) |  | |  |
| No | 1978 | | 0.014 (0.005, 0.023) < 0.01 |
| Yes | 157 | | 0.036 (0.007, 0.066) 0.02 |
| Serum phosphorus, mmol/L |  | |  |
| 0.32-0.97 | 677 | | 0.002 (-0.013, 0.018) 0.77 |
| 0.98-1.14 | 744 | | 0.009 (-0.006, 0.023) 0.26 |
| 1.15-1.90 | 714 | | 0.029 (0.014, 0.043) < 0.01 |
| TC, mmol/L |  | |  |
| 1.34-3.76 | 706 | | 0.005 (-0.009, 0.019) 0.49 |
| 3.77-4.58 | 715 | | 0.028 (0.012, 0.044) < 0.01 |
| 4.59-7.96 | 714 | | 0.009 (-0.007, 0.025) 0.27 |
| Triglycerides, mmol/L |  | |  |
| 0.19-0.79 | 692 | | 0.010 (-0.005, 0.024) 0.20 |
| 0.79-1.24 | 728 | | 0.007 (-0.008, 0.022) 0.36 |
| 1.25-15.46 | 715 | | 0.030 (0.015, 0.046) < 0.01 |
| AST, U/L |  | |  |
| 8-18 | 646 | | 0.015 (-0.000, 0.030) 0.06 |
| 19-24 | 745 | | 0.021 (0.006, 0.037) 0.01 |
| 25-266 | 744 | | 0.008 (-0.007, 0.022) 0.32 |

Adjusted for age; gender; BMI; hypertension; diabetes; heart diseases; CKD; alcohol consumption; smoking status; serum phosphorus; total cholesterol; triglycerides; AST.

Abbreviations: SIRI, systemic inflammation response index; BMI, body mass index; CKD, chronic kidney disease; TC, total cholesterol; AST, aspartate aminotransferase.
