# Supplementary material for: Beyond linearity: a threshold effect links serum creatinine to SIRI in osteoporotic fractures
Source: Front Med (Lausanne). 2025 Dec 18;12:1710691. doi: 10.3389/fmed.2025.1710691 (PMC12756718; doi:10.3389/fmed.2025.1710691)
Supplement: Supplementary file 2 [file Table_2.docx]

Table S2: Regression Results for Different Creatinine Thresholds

| Threshold | Model 1^a^  β (95% CI) | *P*-value | Model 2^b^  β (95% CI) | *P*-value |
| --- | --- | --- | --- | --- |
| Baseline Model | 0.017 (0.008, 0.025) | < 0.01 | 0.013 (0.004, 0.021) | < 0.01 |
| Normal Upper Limit |  |  |  |  |
| 110% | 0.018 (0.009, 0.027) | < 0.01 | 0.014 (0.005, 0.023) | < 0.01 |
| 120% | 0.017 (0.008, 0.025) | < 0.01 | 0.013 (0.004, 0.021) | < 0.01 |
| 130% | 0.017 (0.008, 0.025) | < 0.01 | 0.013 (0.004, 0.021) | < 0.01 |

^a^ Adjusted for age; gender; BMI.

^b^ Adjusted for age; gender; BMI; hypertension; diabetes; heart diseases; CKD; alcohol consumption; smoking status; serum phosphorus; total cholesterol, triglycerides; AST.

Abbreviations: SIRI, systemic inflammation response index; BMI, body mass index; CKD, chronic kidney disease; AST, aspartate aminotransferase.
