# Supplementary material for: Beyond linearity: a threshold effect links serum creatinine to SIRI in osteoporotic fractures
Source: Front Med (Lausanne). 2025 Dec 18;12:1710691. doi: 10.3389/fmed.2025.1710691 (PMC12756718; doi:10.3389/fmed.2025.1710691)
Supplement: Supplementary file 3 [file Table_3.docx]

Table S3 Cox proportional hazards estimate for serum creatinine and SIRI across robustness analyses

A. Linear model

| Scenario | β (95% CI) | *P*-value |
| --- | --- | --- |
| Main analysis ^a^ | 0.013 (0.004, 0.021) | < 0.01 |
| Main analysis (Including Frailty Index) ^b^ | 0.014 (0.005, 0.023) | < 0.01 |
| Excluding acute diseases or stress-related inflammatory conditions ^c^ | 0.013 (0.006, 0.020) | < 0.01 |

B. Piecewise model

| Scenario | Segment ≤78: β (95% CI) | Segment >78: β (95% CI) |
| --- | --- | --- |
| Main analysis ^a^ | 0.001 (-0.013, 0.014) | 0.033 (0.014, 0.052) |
| Main analysis (Including Frailty Index) ^b^ | -0.002 (-0.017, 0.014) | 0.035 (0.015, 0.054) |
| Excluding acute diseases or stress-related inflammatory conditions ^c^ | 0.006 (-0.006, 0.018) | 0.021 (0.007, 0.035) |

^a^ Adjusted for age; gender; BMI; hypertension; diabetes; heart diseases; CKD; alcohol consumption; smoking status; serum phosphorus; total cholesterol; triglycerides; AST.

^b^ Adjusted for age; gender; BMI; hypertension; diabetes; heart diseases; CKD; alcohol consumption; smoking status; serum phosphorus; total cholesterol; triglycerides; AST ;and frailty index.

^c^ Individuals with acute diseases or stress-related inflammatory conditions during the index hospitalization were excluded.

Abbreviations: SIRI, systemic inflammation response index; BMI, body mass index; CKD, chronic kidney disease; AST, aspartate aminotransferase.
